# Characteristics of Tinnitus in Patients Affected by COVID-19: A Systematic Review

**DOI:** 10.1055/s-0045-1802968

**Published:** 2025-08-20

**Authors:** Daniella Wuttke Doutrelepont, Carolina Pereira Fernandes, Luiza Silva Vernier, Marcia Salgado Machado

**Affiliations:** 1 Speech Therapy Course, Universidade Federal de Ciências da Saúde de Porto Alegre (UFCSPA), Porto Alegre, RS, Brazil

**Keywords:** COVID-19, tinnitus, audiology, characteristics

## Abstract

**Introduction:**

The severe acute respiratory syndrome coronavirus 2 (SARS-CoV-2) pandemic has brought up reports of an increase in new cases of tinnitus and changes in chronic and/or preexisting episodes. Nevertheless, there is no established data on the characteristics of tinnitus and its correlation with coronavirus disease 2019 (COVID-19).

**Objective:**

To analyze the characteristics of tinnitus in subjects affected by COVID-19 while detailing the correlation between these two factors.

**Data Synthesis:**

We found 327 articles, 37 of which were selected: 11 cross-sectional studies, 4 case-control studies, 3 cohort studies, and 19 observational studies. The sum of the samples totaled 399,524 patients included in the present review. The prevalence of new tinnitus varied from 0.2% to 96.2%. Most articles provided incomplete information or were missing information. Systemic arterial hypertension was the most common underlying disease. Finally, we found a predominance of hearing loss and olfactory and taste disorders, followed by fever and cough.

**Conclusion:**

The prevalence of new tinnitus ranged from 0.2 to 96.2%, whereas the prevalence of preexisting tinnitus varied from 8 to 76.2%. It was not possible to satisfactorily assess the characteristics of tinnitus. Therefore, a direct correlation between tinnitus and COVID-19 could not be determined, as this symptom may be influenced by other factors.

## Introduction

In March 2020, the World Health Organization (WHO) declared a coronavirus disease 2019 (COVID-19) pandemic. As of August 27, 2021, 215,900,900 confirmed cases have been reported in 223 countries, including 4,494,855 deaths worldwide. 1 The symptoms related to this disease are diffuse, but there is evidence of a correlation between virus contamination and changes in the auditory and vestibular systems, producing symptoms such as tinnitus, 1 2 3 4 5 6 7 8 9 10 11 12 13 14 15 16 17 18 19 20 21 22 23 24 25 26 27 28 29 30 31 32 33 34 35 36 37 38 hearing loss, 1 2 3 4 6 7 9 10 11 12 13 14 16 17 19 20 22 23 24 25 26 27 28 29 30 31 32 33 34 37 38 dizziness/vertigo, 1 2 3 6 8 9 10 11 12 14 16 18 19 21 23 24 29 30 31 32 33 35 36 38 and other manifestations.

In this context, tinnitus stands out, since it is a symptom that impacts quality of life, interfering with sleep and social activities, as well as causing emotional disruptions. 39 Tinnitus occurs more frequently among older adults, which is the age group with highest risk of serious illness caused by COVID-19. 4 Therefore, the severe acute respiratory syndrome coronavirus 2 (SARS-CoV-2) pandemic has generated reports of new cases and changes in chronic and/or preexisting episodes. 1 2 3 4 5 6 7 8 9 10 11 12 13 14 15 16 17 18 19 20 21 22 23 24 25 26 27 28 29 30 31 32 33 34 35 36 37 38

Although this symptom has been observed in several recent studies, there is still the need to detail the characteristics of tinnitus and investigate its correlations with COVID-19 to assess if there are direct connections between both factors. Given the information heterogeneity and the need for in-depth research on the subject, the present study intends to analyze the characteristics of tinnitus in subjects affected by COVID-19, as well as to detail the correlation between these two factors.

## Literature Review

For the search, we used the Health Sciences Descriptors (Descritores em Ciências da Saúde, DeCs, in Portuguese) and Medical Subject Headings (MeSH) filters, as well as the Boolean operators “OR” and “AND”. The electronic databases chosen were PubMed (Medical Literature Analysis and Retrieval System Online, MEDLINE), Web of Science, Latin American and Caribbean Health Sciences Literature (Literatura Latino-Americana e do Caribe em Ciências da Saúde, LILACS, in Portuguese) and Scientific Electronic Library Online (SciELO), with the strategy (without filters): *Covid-19* OR *SARS-CoV-2* OR *Ad26COVS1* OR *ChAdOx1 nCoV-19* AND *Tinnitus* .

The inclusion criteria were free articles written in Portuguese, English or Spanish, with no date or design restrictions, on tinnitus (not necessarily present), whether associated with hearing and/or vestibular complaints and describing any characteristic of tinnitus during/after SARS-CoV-2 contamination. And the exclusion criteria were studies on tinnitus unrelated to SARS-CoV-2 infection, literature reviews, case reports/series, letters to the editor, and academic abstracts.

The search was conducted in three stages (analysis of titles and abstracts, full-text reading of the articles, and data extraction) between August and September 2022, and it was updated in July 2023. The studies were assessed according to the Oxford Center for Evidence-Based Medicine's quality of evidence classification 40 and the Grading of Recommendations Assessment, Development and Evaluation (GRADE) system. 41 All stages were completed by three independent researchers and a judge, and any disagreements were resolved by consensus.

The present research has been registered on the International Prospective Register of Systematic Reviews (PROSPERO; under number CRD42022367295) using the Preferred Reporting Items for Systematic Reviews and Meta-Analyses (PRISMA) statement. 42

## Summary of Search Results

**Fig. 1:**
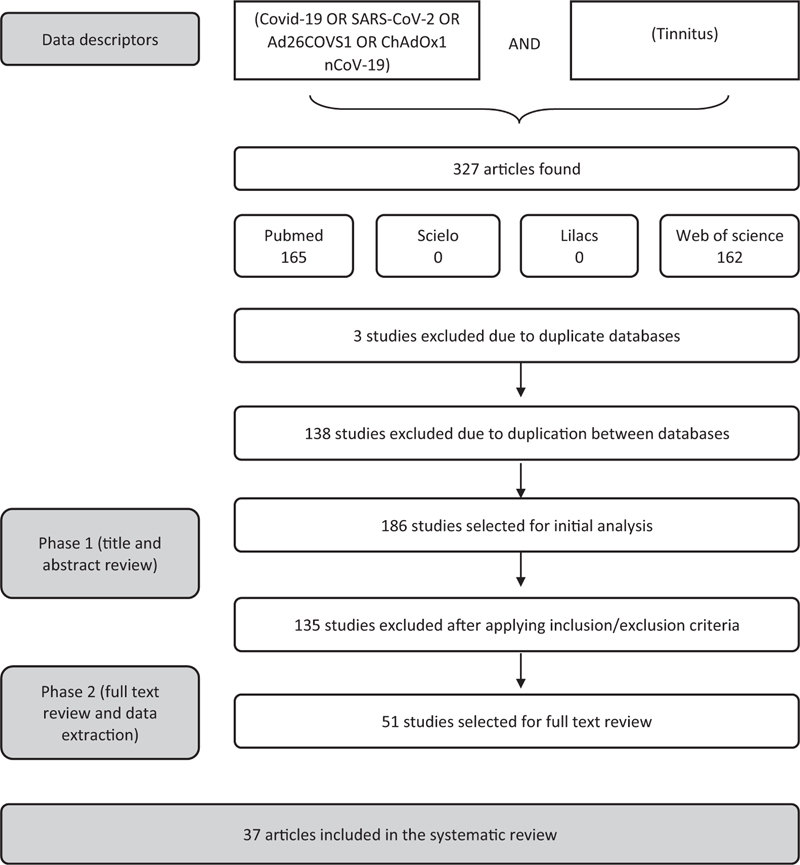
Flowchart of the process of study selection.

We found 327 articles, 37 of which were included in the review. The results from each stage of the selection process, as well as the complete search strategy, can be found in Figure 1 .

The articles selected had the following designs: 11 cross-sectional studies, 4 case-control studies, 3 cohort studies, and 19 observational studies, with the samples resulting in an aggregate number of 399,524 patients included in the present review.

Regarding the quality of the evidence, the articles were assessed based on the Oxford Center for Evidence-based Medicine's quality of evidence classification, 40 which divides studies into five levels, considering their research design and methodology. Concerning the GRADE system, 41 all studies were classified in category C, with a low level of evidence, as they were observational studies. The articles are thoroughly analyzed in Table 1 .

**Table 1:** Classification of the quality of evidence according to the Oxford Center for Evidence-Based Medicine

Study	Level 1	Level 2	Level 3	Level 4	Level 5
Freni et al. 27				X	
Liang et al. 15				X	
Elibol 17				X	
Özçelik Korkmaz et al. 16		X			
Viola et al. 21				X	
Beukes et al. 4				X	
Gallus et al. 3				X	
Bhatta et al. 20			X		
Gosavi et al. 7				X	
AlJasser et al. 12			X		
Hassani et al. 24		X			
Yaseen et al. 6				X	
Çıldir 2				X	
Ferreira et al. 23				X	
Deva et al. 14				X	
Verma et al. 25				X	
Saunders et al. 26				X	
Almishaal and Alrushaidan 19				X	
Degen et al. 18				X	
Ali et al. 8			X		
Zięba et al. 9				X	
Thrane et al. 11				X	
Saraf et al. 5				X	
Kartal and Kılıç 22				X	
Erinc et al. 13			X		
Espinoza-Valdez et al. 10				X	
Figueiredo et al. 28				X	
Khanna et al. 38				X	
Aldè et al. 30				X	
Almishaal 32				X	
Africa et al. 34				X	
Kwaśniewska et al. 33				X	
Elmazny et al. 36				X	
Bilgin et al. 35				X	
Dorobisz et al. 37			X		
Borda Pedraza et al. 31				X	
Wu et al. 29				X	

The selected studies provide multiple data related to SARS-CoV-2 infection and tinnitus. These comprise the symptom's prevalence in cases of infection by the virus, manifesting in the most diverse ways, ranging from 0.2 35 to 96.2%. 6 Moreover, the number of samples and the methodology used in each article vary according to the study design. Of these, 13 studies 4 5 8 12 13 20 24 26 30 31 32 34 37 included control groups, that is, individuals not infected and/or who did not report being infected with SARS-CoV-2 to compare with the complaints reported by those affected by the disease. Among those selected, 11 articles 4 5 12 13 16 19 22 23 26 27 28 provide information related to changes in preexisting tinnitus, that is, changes (positive or negative) reported by patients regarding this symptom after COVID-19 infection. The characteristics of the selected articles, the tinnitus prevalence, and the main conclusions are presented in Table 2 .

**Table 2:** Characterization of the selected studies, prevalence of tinnitus and main conclusions

Study	Year	GRADE	Design	Sample (n)	Age (years)	Tinnitus prevalence (COVID positive)	Conclusions
Freni et al. 27	2020	C	Observational longitudinal	50	18–65; mean: 37.7 ± 17.9	20% (onset or worsening)	There were changes in taste and smell, dry eyes, and oral cavity and hearing discomfort in the studied population, symptoms probably tied to the neurotropism aspect of the virus.
Liang et al. 15	2020	C	Observational	86	06–57; median: 25.5	3.5%	Sensorineural dysfunction is most likely to occur in the early stages of COVID-19 and may be used as a marker for early diagnosis of the disease.
Elibol 17	2021	C	Retrospective observational	155	18–72; mean: 36.3 ± 8.1	1.2%	Tinnitus, gingivitis, sudden HL, Bell's palsy and hoarseness can be seen in COVID-19, albeit rarely. Revealing ENT symptoms of COVID-19 and finding out more information on the extent of the disease will be useful in managing patients and their complaints.
Özçelik Korkmaz et al. 16	2021	C	Observational prospective cohort	116	19–83; mean: 57.24 ± 14.32	11.2%	Patients infected with SARS-CoV-2 may have different symptoms in otologic or laryngeal areas. More careful assessments should be made of ENT symptoms when SARS-CoV-2 infection is suspected.
Viola et al. 21	2020	C	Observational multicenter	185	19–81; mean: 52.15 ± 13	23.2%	Patients with COVID-19 may report subjective otoneurological symptoms, such as tinnitus and balance disorders. More studies are needed to investigate the prevalence and pathophysiological mechanisms underlying these symptoms.
Beukes et al. 4	2020	C	Observational cross-sectional	3,103; 26/3,103 COVID-19 +	18–100; mean: 58 ± 14.0	8%; divided into: ● 54% (stable tinnitus); ● 6% (better tinnitus); ● 40% (significantly worse tinnitus)	The findings have implications for tinnitus treatment, as they prove the diverse response that internal and external factors have on tinnitus levels. Clinical services should be aware that tinnitus can be caused by SARS-CoV-2 virus, just as preexisting tinnitus can be exacerbated.
Gallus et al. 3	2021	C	Retrospective observational	48	average: 45	4.2%	Audiovestibular symptoms are mostly transient and there is no clear-cut evidence of clinically persistent or relevant cochlear or vestibular damage after recovery.
Bhatta et al. 20	2022	C	Observational prospective case-control	331 COVID-19 + and 10 control groups from each institute (8 institutes = 80)	Study group: 18–38; mean: 32 ± 4.3	1.8%	There was no relevant difference in the hearing status of COVID-19-positive patients compared to the control group. There were changes in the functioning of the Eustachian tube and middle ear in those infected with SARS-CoV-2.
Gosavi et al. 7	2022	C	Observational prospective	70	10–70; Mean: 50.35 ± 17.41	3.17%	Knowing the ENT symptoms in COVID-19 patients is helpful to conduct an early quarantine and limit viral transmission. It can also act as an important tool for a faster start of COVID-19 therapy.
AlJasser et al. 12	2022	C	Observational case-control	300 COVID -19 +; 150/300 isolated; 150/300 hospitalized; 267 COVID - 19 -; 150/267 Isolated; and 117/267 hospitalized	Positive cases: hospitalized – 19–65; average: 44.2; isolated at home: 18–64; average: 35.9	8% (hearing deterioration and/or tinnitus – COVID-hospital); 8% (hearing deterioration and/or tinnitus – COVID-home); 2% (recent changes in hearing and tinnitus – COVID-hospital); 0.7% (changes in hearing and tinnitus – COVID-home); 5.3% (changes in tinnitus – COVID- hospital); and 4.7% (changes in tinnitus – COVID-home)	There is no evidence that COVID-19 leads to a deterioration of hearing or tinnitus during the acute phase or after recovery, in mild or severe cases.
Hassani et al. 24	2021	C	Observational prospective cohort	57 patients; 31/57: COVID-19 +; and 26/57: no history of COVID-19	COVID-19 + patients: 21-50; mean: 33.87 ± 9.85	6.45%	In mild to moderate cases of the disease, ear symptoms vanish within a week and the virus has no lasting impact on the auditory system.
Yaseen et al. 6	2021	C	Retrospective observational	26	21–66; mean: 39.23 ± 11.884	96.2%	Most COVID-19-related SSNHL cases occurred within a week of the infection onset, with a predominance of bilateral cases. Tinnitus was the most common symptom.
Çıldir 2	2021	C	Retrospective observational	1,437	18–80; mean: 40.6 ± 14.3	0.9% (onset on the 2nd day of illness) . 19.4% (14th day of illness)	Symptoms such as dizziness, tinnitus and decreased sound tolerance can be seen in the last days of COVID-19.
Ferreira et al. 23	2021	C	Observational, descriptive, and quantitative	173	18–72; mean: 35.4 ± 11.4	14.5% (before COVID-19 confirmation); 43.9% (after COVID-19 confirmation)	High occurrence and worsening of auditory and vestibular symptoms after COVID-19.
Deva et al. 14	2022	C	Retrospective observational	286	18–60; mean: 36.3 ± 8.1	7.4% (tinnitus); 14.6% (vertigo and tinnitus); 20.3% (tinnitus and HL) 6.25% (tinnitus, vertigo, and HL)	This study proves the different array of manifestations of COVID-19, including vertigo, tinnitus, and HL.
Verma et al. 25	2022	C	Observational	78	18–59; mean: 35.78 ± 11.93	66.66%	Individuals with comorbidities, admitted to the ICU for COVID-19 treatment, with prolonged hospitalization, were at a greater risk of developing problems related to speech, swallowing, and hearing after COVID-19.
Saunders et al. 26	2022	C	Observational cross-sectional	6;881; 411/6,881 (6%) COVID-19 confirmed (COVID + ); 760/6,881 (11%) probably had COVID-19 (COVID-P); 5,710/6,881 (83%) did not have COVID-19 (COVID-0)	Mean (total sample): 52.1 ± 16.1; mean (COVID +): 45.4 ± 15.2; and mean (COVID-P): 46.4 ± 14.6	16.3% (COVID +); 16.6% (COVID-P); 23.1% (COVID +) – worsening of preexisting tinnitus; 22.8% (COVID-P) – worsening of preexisting tinnitus; 10.2% (total): hearing difficulties and tinnitus; and 11.7% (total): tinnitus only	Notwithstanding the fact that there are more reports of hearing symptoms from individuals with confirmed or probable COVID-19, there are some inconsistent reports and/or memory bias, as well as possible placebo effects. Studies that include appropriate control groups and rely on audiometric measures, besides self-reporting to investigate changes in hearing symptoms compared to pre-COVID-19, are urgently needed.
Almishaal and Alrushaidan 19	2022	C	Observational cross-sectional	301	27–44; mean: 36.58 ± 12.54	2.99% (before SARS-CoV-2 contamination); 9.97% (during the COVID-19 acute phase); and 1.66% (6 months after SARS-CoV-2 infection)	Audiovestibular symptoms are common among patients infected with SARS-CoV-2 during the acute phase. These symptoms are mainly temporary, with complete spontaneous recovery during the first 2 weeks postinfection.
Degen et al. 18	2022	C	Observational	1,082	18–70 + ; mean: 43.0 ± 12.4	30.6%	Tinnitus, vertigo, and dizziness are common symptoms in patients with long COVID-19, revealing that a convincing number of patients classify their symptoms as severe.
Ali et al. 8	2022	C	Observational case-control	100, 52 of whom completed the follow-up study. Of these, 27/52 COVID-19 + and 25/52 COVID-19 -	Mean: 42.8 ± 11.5	19% (tinnitus at first visit); 26% (tinnitus at follow-up visit)	*Long haulers* not hospitalized with COVID-19 continue to have neurological symptoms, fatigue and lower quality of life 14.8 months after the initial infection.
Zięba et al. 9	2022	C	RetrospectiveoObservational	337	18–86; mean: 43.98 ± 13.47	15%	Delayed diagnosis and treatment of symptoms, particularly those related to the hearing organ, can result in more permanent damage.
Thrane et al. 11	2022	C	Retrospective observational	225	19–76; average: 45.5	6.4% (database); 67.7% (follow-up questionnaire)	A relevant share of the population had concomitant, long-lasting audiological symptoms with a negative impact on quality of life. Further research is needed into the association between COVID-19 and audiovestibular symptoms and the need for rehabilitation among convalescents.
Saraf et al. 5	2022	C	Retrospective observational	90; 21/90 COVID-19 +	20–65 +; mean: 39.06 ± 7.66	80% (long-term chronic tinnitus); 20% (postinfection tinnitus). Changes in pre-existing tinnitus: ● 76.2% (tinnitus exacerbation); ● 19% (stable tinnitus); and ● 4.8% (better tinnitus)	Otolaryngologists must be fully aware that pre-existing tinnitus may not only be exacerbated due to SARS-CoV-2 infection, but there can also be development of new-onset tinnitus due to COVID-19 involvement.
Kartal and Kılıç 22	2023	C	Observational cross-sectional	279	18–60	28% divided into: ● 12.20% (pre-existing tinnitus, worsening during COVID-19); ● 10% (tinnitus having started alongside COVID-19 treatment); and ● 5.70 (tinnitus started after recovery from COVID-19)	Doctors should keep in consideration that tinnitus can be caused by COVID-19, just as preexisting tinnitus can be exacerbated by it. Nevertheless, the majority of study participants did not experience post-COVID-19 tinnitus.
Erinc et al. 13	2022	C	Observational cohort	96; 37/96 COVID-19 +; and 59/96 COVID-19 -	20–83; mean: 50.8 ± 14.5	5/96 patients – worse tinnitus during COVID-19, remaining worse afterwards; 3/96–worse tinnitus during COVID-19, with recovery afterwards; 1/96–worsening of tinnitus during and after COVID-19; 0% (decreased tinnitus); 24.3% (increased tinnitus); and and 75.7% (no change)	This study mentions different effects of SARS-CoV-2 infection and the pandemic period on patients with chronic tinnitus. It also provides evidence for the deterioration of preexisting tinnitus as a possible long-term effect of COVID-19.
Espinoza-Valdez et al. 10	2022	C	Observational, cross-sectional, descriptive, and analytical	209	20–60 +	43.5%	The most common otoneurological manifestations were vertigo, tinnitus, and imbalance. The clinical traits associated with an otoneurological profile were asthenia, hyposmia, and dysgeusia.
Figueiredo et al. 28	2022	C	Observational cross-sectional	57; 33/57 (57.9%) without tinnitus; 13/57 (22,8%) pre-COVID-19 tinnitus; 11/57 (19.3%) post-COVID-19 tinnitus	≥ 18; group without tinnitus: median – 45; Pre-COVID tinnitus group: median – 58; Post-COVID tinnitus group: median – 53	69.2% (stable tinnitus after SARS-CoV-2 infection); 30.8% (worsening after SARS-CoV-2 infection)	There was no difference in disease symptoms or treatment between COVID-19 patients who developed tinnitus and those who did not. Generally speaking, tinnitus emerging within a COVID-19 infection context does not seem to differ from tinnitus unrelated to COVID-19.
Khanna et al. 38	2022	C	Observational, cross-sectional, and retrospective	592	13–60; average: 34.37	7.24%	Otorhinolaryngological symptoms are fairly common in patients with mild and early COVID-19, and a high rate of suspicion of COVID-19 in patients with symptoms of otorhinolaryngology should be analyzed.
Aldè et al. 30	2023	C	Retrospective observational	272 (132 COVID + and 140 COVID -)	5-11 years Average 7.8 (± 2.3) years	8.30%	This study asserts the safety of the COVID-19 vaccine regarding the audiovestibular system. It also shows that a SARS-CoV-2 infection can cause audiovestibular symptoms in a high percentage of the pediatric population.
Almishaal 32	2022	C	Observational cross-sectional	939 (120 COVID - and 794 COVID +); 301 Alpha/Beta; 102 Delta; and 416 Omicron	> 18; average: 36	10.4%: total; 1.7%: controls; 10%: Alpha/Beta; 13.7%: Delta; 12.5%: Omicron	Audiovestibular symptoms are commonly encountered during the acute phase of the Alpha/Beta, Delta, and Omicron variants of COVID-19. These findings show the need for otorhinolaryngologists and speech therapists to conduct physical, behavioral, and electrophysiological audiovestibular tests to assess the possible etiopathology of audiovestibular dysfunction associated with COVID-19.
Africa et al. 34	2023	C	Observational	187,587 COVID + and 187,587 COVID -	18–90	0.08%: 5 days after diagnosis; 0.11%: 10 days after diagnosis; 0.10%: 15 days after diagnosis; and 0.13%: 1 month after diagnosis	Tinnitus and vestibular disorders may not be clinically significant due to their low incidence.
Kwaśniewska et al. 33	2023	C	Observational	346	18–64; mean: 39.8 ± 9.56	6.07%	There is a statistically significant correlation between some otorhinolaryngological symptoms and the levels of anti-SARS-CoV-2 antibodies in the convalescent plasma.
Elmazny et al. 36	2023	C	Observational cross-sectional	1,638	> 18	8.10%	Neuropsychiatric sequelae found in 36.5% of the Egyptian survivors of COVID-19, with fatigue being the most prevalent.
Bilgin et al. 35	2023	C	Observational p-rospective	1,701	> 18	0.20%	Long-COVID symptoms have been commonly found in outpatients with COVID-19. Age, gender and BMI may be factors that affect prolonged COVID symptoms.
Dorobisz et al. 37	2023	C	Observational case-control	58 COVID + And 60 COVID -	Cases: 23–75 (average: 48); controls: 38–69	77%	COVID-19 can damage the inner ear and the auditory pathway. Hearing loss may be the only symptom of COVID-19, or it can be a late complication of the disease due to postinfectious inflammation of the nerve tissue as a symptom of long COVID-19.
Borda Pedraza et al. 31	2023	C	Observational, cross-sectional, and analytical	62 (40 COVID + and 22 COVID -)	20–45; average: 31.05	17.50%	Audiovestibular symptoms are common in symptomatic patients recovering from SARS-CoV-2 infection, consistently associated with an increase in the audiometric hearing threshold at specific frequencies and a low-tone mean.
Wu et al. 29	2023	C	Observational, cross-sectional, descriptive, and analytical	2.247	Average: 36	19.05%	Otologic symptoms are commonly found among individuals infected with COVID-19 during the acute phase of the pandemic. These symptoms appear, for the most part, to vanish spontaneously. But the true prevalence of cochleovestibular system and facial nerve involvement in COVID-19 patients worldwide remains unknown.

**Abbreviations:** BMI, Body Mass Index; COVID-19, coronavirus disease 2019; COVID-19 +, COVID-19-positive; COVID-19 -, COVID-19-negative; ENT, ear, nose, and throat; GRADE, Grading of Recommendations Assessment, Development and Evaluation; HL, hearing loss; ICU, Intensive Care Unit; SARS-CoV-2, severe acute respiratory syndrome coronavirus 2; SSNHL, sudden sensorial hearing loss.

Regarding the characteristics of tinnitus, only 4 23 28 30 34 studies provided data on symptom onset, and data on this was treated differently among the studies. As for the laterality of the symptom, six articles 5 13 14 24 29 37 reported unilateral or bilateral tinnitus, and two 20 28 reported exclusively bilateral tinnitus. Moreover, information on the characteristics of the sound is scarce, with only four articles reporting it. 14 26 28 29 Details on duration, onset, laterality, acoustic features, and information on periodicity, aggravation, and reversibility of tinnitus are shown in Table 3 .

**Table 3:** Data on the duration, laterality, acoustic characteristics, periodicity, onset, aggravation, and reversibility of tinnitus

**Study**	**Complaint time**	**Emergence**	**Laterality/ Location**	**Sound** **characteristics**	**Frequency**	**Period of emergence**	**Post-COVID aggravation**	**Reversibility**
Freni et al. 27	5/50 (10%) patients had tinnitus for 15 days after COVID-19	Not available	Not available	Not available	Not available	Before and during COVID-19	10/50 (20%) patients had tinnitus onset or worsening	Reversible (in 5/50 cases, tinnitus disappeared before 15 days) and irreversible (5/50 cases maintained the symptom 15 days after the negative test)
Liang et al. 15	Symptoms appeared in 6 ± 5.29 days and lasted an average of 5 ± 0 days	Not available	Not available	Not available	Not available	During COVID-19	Not available	Reversible; mean duration of tinnitus of 5 ± 0 days
Elibol 17	Not available	Not available	Not available	Not available	Not available	After COVID-19 (not specified)	Not available	Not available
Özçelik Korkmaz et al. 16	Complaints lasted a minimum of 1 day and a maximum of 9 days. Average duration of 4 days	Not available	Not available	Not available	Not available	Before and during COVID-19	Not available	Reversible; the complaints lasted a maximum of 9 days
Viola et al. 21	Not available	Not available	Not available	2/43 (4.6%); continuous; 3/43 (7%): pulsating	17/43 (39.5%): recurrent; 10/43 (23.3%): occasional; 7/43 (16.3%): fluctuating; 4/43 (9.3%): persistent	After COVID-19 (31–59 days after COVID-19 diagnosis)	Not available	Not available
Beukes et al. 4	The average duration of tinnitus was of 13.6 years, ranging from 0.3 to 80 years	Not available	Not available	Not available	Not available	Before and after COVID-19	95/237 (40%) – self-reported COVID-19 patients had exacerbated tinnitus; 15/26 (58%) – COVID-19 + patients had their tinnitus worsen	Not available
Gallus et al. 3	Not available	Not available	Not available	Not available	Not available	After COVID-19	Not available	1/2 case irreversible and 1/2 reversible
Bhatta et al. 20	Less than 3 months	Not available	Bilateral	Not available	Not available	During COVID-19 (onset within 14 days of positive test)	No	Reversible (disappearance of the complaint 3 months postinfection)
Gosavi et al. 7	Not available	Not available	Not available	Not available	Not available	During COVID-19	Not available	Not available
AlJasser et al. 12	Not available	Not available	Not available	Not available	Not available	Before, during, and after COVID-19	COVID-home: 7/150 reported deterioration; COVID-hospital: 8/150 reported deterioration; there was also deterioration in the control group	Reversible; 1.3%: COVID-hospital; 2.7%: COVID-home
Hassani et al. 24	Not available	Not available	½: unilateral (on the right); ½: bilateral	Not available	Not available	During COVID-19	No	Irreversible
Yaseen et al. 6	Not available	Not available	Not available	Not available	Not available	During COVID-19	Not available	Not available
Çıldir 2	Onset on the 2nd day of diagnosis, ending 14 days later	Not available	Not available	Not available	Not available	During COVID-19	Not available	Reversible (data show remission after 3 months)
Ferreira et al. 23	Not available	49/173 (28.3%): progressive; 43/173 (24.9%): regressive; 22/173 (12.7%): Immediate emergence. Note: information in relation to symptoms in general	Not available	Not available	Not available	25/173 (14.5%) before COVID-19; 76/173 (43.9%) after COVID-19	The percentage of patients complaining of tinnitus went from 14.5% (before the COVID-19) to 43.8% (after COVID-19)	107/173 (61.8%) with symptoms were stable and present up to the time of the survey, and 66/173 (38.2%) reported that the manifestation was temporary and disappeared with time
Deva et al. 14	Not available	Not available	3/21 (14.2%): bilateral; 18/21 (85,7%): unilateral	11/21 (52.3%) had mild tinnitus (silence situation); 7/21 (33.3%) had moderately-loud tinnitus (normal situation); and 3/21 (14.2%) had loud tinnitus	17/21 (81%): episodic tinnitus; 4/21 (19%): persistent tinnitus	Not available	Not available	Not available
Verma et al. 25	Not available	Not available	Not available	High frequency	Not available	After COVID-19	No	Irreversible
Saunders et al. 26	Not available	Not available	Not available	Not available	Not available	25% before COVID-19; 38.9% during/after COVID-19	23.1% (COVID +): worsening of tinnitus; 22.8% (COVID-P): worsening of tinnitus	Not available
Almishaal and Alrushaidan 19	The mean duration of hearing symptoms was of 7.8 ± 16.5 days; 1,66% of participants reported tinnitus after 6 months of infection	Not available	Not available	Not available	Not available	2.99% prior to COVID-19; 9.97% during COVID-19	11.63% of participants reported that their symptoms worsened after SARS-CoV-2 infection. Note: there was improvement of hearing symptoms in general, but with no specific information on tinnitus	Irreversible after 6 months for 1.66% of the patients
Degen et al. 18	Not available	Not available	Not available	Not available	Not available	After COVID-19	Not available	Due to the lack of follow-up, it remains unclear whether the severity of these symptoms has decreased or remained constant over time
Ali et al. 8	Not available	Not available	Not available	Not available	Not available	During and after COVID-19	Not available	Not available
Zięba et al. 9	15 days	Not available	Not available	Not available	Not available	During and after COVID-19	Not available	In 48% of the cases, the tinnitus was resolved completely, and in 21%, partially
Thrane et al. 11	7/225 declared that the symptom was still present during the 258.8 days of the survey	Not available	Not available	Not available	Not available	After COVID-19	Not available	7/21 patients reported recovery, 7/21 reported a reduction in symptoms, and 7/21 reported no reduction in tinnitus
Saraf et al. 5	The mean duration of tinnitus was of 2.22 ± 0.83 years	Not available	31/90 (34.4%): bilateral; 59/90 (65.5%): unilateral	Not available	Not available	Before and during COVID-19	16/21 (76.2%) patients reported a history of worsening tinnitus	1/16 (4.7%) patient reported improvement in tinnitus after COVID-19
Kartal and Kılıç 22	Not available	Not available	Not available	Not available	Not available	34/279 (43.6%): before; 28/279 (35,9%): during; and 16/279 (20,5%): after COVID-19	34/279 (43.6%) had tinnitus before COVID-19 and worsened during the disease	Not available
Erinc et al. 13	COVID-19 + patients (mean duration): 75 ± 63,4 (range: 27–384) months	Not available	53/96 (55.2%): bilateral; 15/96 (15.7%): right; 28/96 (29.1%): left	Not available	Not available	Before COVID-19	COVID-19 + group: none of the patients reported a decrease in tinnitus discomfort; 9/37 (24.3%) patients reported an increase in tinnitus complaints; and 28/37 (75.7%) did not change.	Irreversible; patients with chronic tinnitus. In the COVID-19 group, none of the patients reported a decrease in tinnitus-related discomfort
Espinoza-Valdez et al. 10	Not available	Not available	Not available	Not available	Not available	During COVID-19	Not available	Not available
Figueiredo et al. 28	Patients with chronic tinnitus = median duration of 36 months. Patients with post-COVID tinnitus = median of 1.5 months	Sudden onset more prevalent in patients with post-COVID tinnitus and gradual onset in patients with chronic tinnitus	Bilateral more prevalent in both groups	30.8% of the group with pre-COVID tinnitus: whistling; 45.5% of the group with post-COVID tinnitus: hissing; continuous/ constant sound in groups with chronic tinnitus post-COVID; and pulsatile: 1 case	Not available	Before and after COVID-19	Among the patients with chronic tinnitus, in 9/13 (69.2%), there was no worsening, while 4/13 (30.8%) reported worsening	Not available
Khanna et al. 38	101/592 (48.79%): 2 weeks; 83/592 (40.09%): 2–4 weeks; 23/592 (11.11%): 1 month (all symptoms); 5/592 (2.41%): 6 months	Not available	Not available	Not available	Not available	After COVID-19	Not available	Reversible
Aldè et al. 30	Not available	9/132 (81.8%): up to 2 weeks; 2/132 (18.2%): 2–4 weeks	Not available	Not available	Not available	During and after COVID-19	Not available	Not available
Almishaal 32	Not available	Not available	Not available	Not available	Not available	After COVID-19	Not available	Not available
Africa et al. 34	Not available	5 days; 10 days; 15 days or 1 month after diagnosis	Not available	Not available	Not available	During and after COVID-19	Not available	Not available
Kwaśniewska et al. 33	Not available	Not available	Not available	Not available	Not available	Not available	Not available	Not available
Elmazny et al. 36	At least 2 months	Not available	Not available	Not available	Not available	During and after COVID-19	Not available	Not available
Bilgin et al. 35	Not available	Not available	Not available	Not available	Not available	After COVID-19	Not available	Not available
Dorobisz et al. 37	Not available	Not available	43.1%: unilateral; 32.8%: bilateral	Not available	Not available	After COVID-19	Not available	Not available
Borda Pedraza et al. 31	Average duration of 14 days (all symptoms)	Not available	Not available	Not available	Not available	After COVID-19	Not available	Not available
Wu et al. 29	Not available	Not available	224/2,247 (52.34%): unilateral; 204/2,247 (47.66%): bilateral	164/2,247 (38.32%): low Frequency; 121/2,247 (28.27%): high Frequency; 143/2,247 (33.41%): did not know how to define it	162/2,247 (37.85%): occasional, intermittent, and tolerable; 86/2,247 (20.09%): continuous and tolerable; 7/2,247 (1,64%): intolerable.	Not available	Not available	184/2,247 (42.99%): total resolution; 179/2,247 (41.82%): partial resolution; 65/2,247 (15.19%): persistent

**Abbreviations:** COVID-19, coronavirus disease 2019; COVID-19 +, COVID-19-positive; COVID-19 -, COVID-19-negative; COVID–P, probably-positive COVID patients; SARS-CoV-2, severe acute respiratory syndrome coronavirus 2.

As for the characteristics of the participants, the results revealed comorbidities in the subjects of 17 articles, 2 6 7 10 13 15 16 17 19 23 25 27 29 31 32 33 34 with preexisting systemic arterial hypertension (SAH) being the most common underlying disease, present in 26/1,437, 2 1/26, 6 19/70, 7 30/209, 10 13/96, 13 2/86, 15 23/116, 16 28/155, 17 35/301, 19 14/173, 23 1/78, 25 8/50, 27 127/2,247, 29 81/939, 32 9/346, 33 and 24/187,587 34 individuals, totaling 16 studies included in this review. Likewise, in 12 articles, 6 7 9 13 16 19 23 25 27 29 32 34 diabetes mellitus was found in 1/26, 6 24/70, 7 3/96, 13 13/116, 16 39/301, 19 5/173, 23 3/50, 27 41/2247, 29 81/939, 32 and 11/187,587 34 subjects. However, two studies 9 25 did not detail the number of patients affected by this underlying disease.

Thirteen articles 7 12 13 15 16 17 18 19 22 23 25 31 38 also stated that the individuals in their samples required hospitalization due to SARS-CoV-2 infection. Specifically, outpatient care was required in 70/70, 7 150/300, 12 4/96, 13 86/86, 15 116/116, 16 64/155, 17 80/1,082, 18 103/301, 19 40/279, 22 5/40, 31 and 592/592 38 patients, whereas the other studies 23 25 that present data on this did not provide the number of subjects affected. Nonetheless, in 1 of these cases, 25 2/78 individuals required hospitalization in the Intensive Care Unit (ICU). Only 12 studies presented data related to the medications used by patients 4 5 8 10 19 22 23 25 28 29 31 32

Finally, all of the studies reported at least one piece of information related to other auditory and extra-auditory symptoms, 2 3 4 6 7 8 9 10 11 12 13 14 15 16 17 18 19 20 21 22 23 24 25 26 27 28 29 30 31 32 33 34 35 36 37 38 except one, 5 which focuses exclusively on tinnitus. Details of the characteristics of the participants regarding COVID-19 and auditory and extra-auditory symptoms can be found in Table 4 .

**Table 4:** Characteristics of participants regarding SARS-CoV-2 contamination and auditory and extra-auditory symptoms

**Study**	**Comorbidities**	**Hospitalization due to COVID-19**	**Medication for COVID-19**	**Auditory/vestibular symptoms**	**Extra-auditory symptoms**
Freni et al. 27	14/50 (28%): gastroesophageal reflux disease; 3/50 (6%): diabetes; 8/50 (16%): hypertension; 5/50 (10%): congenital rubella syndrome; 4/50 (8%): thyroid disease; 10/50 (20%): allergies; 7/50 (14%): asthma; 2/50 (4%): heart issues; and 4/50 (8%): autoimmune diseases	Not available	Not available	Hearing loss	Cough, asthenia, headache, nausea/vomiting, abdominal pain, diarrhea, myalgia, arthralgia, mucus, loss of appetite, fever, nasal obstruction, postnasal drip rhinorrhea, sore throat, facial pain, otalgia, lacrimation changes, dysphagia, dyspnea, dysphonia, olfactory dysfunction, xerostomia, dry eye, gustatory dysfunction, and anosmia
Liang et al. 15	8/86 (9.3%): chronic liver disease; 3/86 (3.5%) hyperlipidemia; 3/86 (3.5%): Heart-brain vascular disease; and 2/86 (2.3%): hypertension, anemia and hyperthyroidism	Yes, all (100%)	Not available	Not available (only tinnitus)	Cough, fever, fatigue, pharyngalgia, anorexia, headache, myalgia, diarrhea, vomiting, hyposmia, and hypogeusia
Elibol 17	28/155 (18%): hypertension; 21/155 (13.5%): asthma; 14/155 (9.6%): cardiovascular disease; and 8/155 (5.1): chronic obstructive pulmonary disease and other comorbidities	Yes (64/155 patients)	Not available	Otalgia and sudden hearing loss	Cough, anosmia, ageusia, sore throat, nasal congestion, postnasal discharge, runny nose, hoarseness, gingivitis, and Bell's Palsy
Özçelik Korkmaz et al. 16	23/116 (19.8%): hypertension; 13/116 (11.2%): diabetes mellitus; 5/116 (4.3%): kidney failure; 7/116 (6%): heart disease; 8/116 (6.9%) chronic respiratory disease/asthma; 14/116 (12.1%): allergic rhinitis; and 8/116 (6.8%): sinonasal issues	Yes, all (100%)	Not available	Dizziness, hearing loss and vertigo	Dry cough, dyspnea, headache, nausea/vomiting, hypogeusia/ageusia, hyposmia/anosmia, sore throat, dysphagia, vocal impairment, pharyngeal globus, nasal obstruction, rhinorrhea, sneezing/runny nose, eye complaints, and high fever
Viola et al. 21	Not available	Not available	Not available	Dizziness and acute bouts of vertigo	Migraine
Beukes et al. 4	Not available	Not available	Ibuprofen, paracetamol, lorazepam, methylprednisolone, mortin, tamiﬂu tablets, tylenol, robitussin, azelastine, salbutamol or azithromycin. No information on dose or time of use	Hearing loss	Not available
Gallus et al. 3	Not available	Not available	Not available	Hearing loss, dizziness, vertigo, static imbalance, dynamic imbalance, intolerance to head movement, and visually-triggered dizziness	Fever, dyspnea, cough, chest pain, asthenia, myalgia, diarrhea, conjunctivitis, general malaise, sore throat, headache, rash, hyposmia, and hypogeusia
Bhatta et al. 20	Not available	Not available	No	Ear fullness, hearing loss and otalgia	Not available
Gosavi et al. 7	19/70 (27.14%): hypertension; 24/70 (34.28%): type-2 diabetes mellitus; 4/70 (5.71%): hypothyroidism; 2/70 (2.85%): heart disease; 3/70 (4.28%): kidney disease; 1/70(1.43%): asthma; 1/70 (1.43%): tuberculosis; and 5/70 (7.14%): other	Yes, all (100%)	Not available	Aural fullness, hearing loss, and otalgia	Sore throat, dysphagia, voice changes, nasal obstruction, rhinorrhea, postnasal discharge, sneezing, headache, facial heaviness, and olfactory and taste dysfunctions
AlJasser et al. 12	Not available	Yes (150/300 COVID-19 +)	Not available	Hearing loss, hyperacusis, dizziness, vertigo, ear pressure, and otalgia	Smell and taste changes, unsteadiness/lightheadedness, fever, difficulty breathing, cough, sore throat, muscle/body pain, headache, and diarrhea
Hassani et al. 24	Not available	Not available	Not available	Hearing loss, ear fullness, otalgia, dizziness, vertigo, and hyperacusis	Communication difficulties, anxiety, insomnia, and severe depression
Yaseen et al. 6	1/26 (3.8%): hypertension; and 1/26 (3.8%): diabetes mellitus	Not available	Not available	Vertigo and hearing loss	Anosmia, dysphonia, and ageusia
Çıldir 2	1/1,437 (0.06%): anemia; 64/1,437 (4,45%): hypertension; 26/1,437 (1.8%): asthma; 19/1437 (1.32%): cardiovascular diseases; and 9/1437 (0.6%): cases of hypothyroidism	No	Not available	**Day 2 of diagnosis:** dizziness, vertigo, difficulty understanding speech in noisy environments, decreased tolerance to sound, otalgia, and ear pressure. **Day 14 of diagnosis:** dizziness, vertigo, difficulty understanding speech in noisy environments, decreased tolerance to sound, otalgia, and ear pressure. **3 months after diagnosis:** dizziness/vertigo continued for a few weeks and then stopped	**Day 2 of diagnosis** : cough, fatigue, dyspnea, chills, arthralgia and notalgia, headache, speech disorder, dysphagia, aphonia, ageusia, anosmia, sore throat, diarrhea, fever, and nasal congestion. **Day 14 of diagnosis** : cough, fatigue, nausea and vomiting, dyspnea, chills, arthralgias and notalgias, headache, speech disorder, dysphagia, aphonia, ageusia, anosmia, sore throat, diarrhea, fever, and nasal congestion. **3 months after diagnosis:** loss of taste, loss of smell, frequent and persistent headache, and dyspnea, especially during rapid movements
Ferreira et al. 23	21/173 (12.1%): respiratory diseases; 11/173(11.3%): gastrointestinal diseases; 8/173 (4.3%): endocrine diseases; 6/173 (3.4%): immunosuppressive diseases; 5/173 (2.8%): neurological diseases; 4/173 (2.3%): cardiopulmonary or cardiovascular diseases: 2/173 (1.1%): hematologic diseases; 1/173 (0.5%): skin diseases; 1/173 (0.5): muscular diseases; 14/173 (8.1): hypertension; and 5/173 (2.8%): diabetes	Yes (4%)	Azithromycin, ivermectin, antihistamine, chloroquine, zinc, dexamethasone, prednisone, vitamins C and D, and dipyrone	Dizziness, “clogged ears”, imbalance, “hearing but not understanding”, hypoacusis, otalgia, difficulty understanding in noise, and otorrhea	Decreased sense of smell, headache, muscle changes, loss of taste, skin problems, olfactory intolerance, fatigue, back pain, body pain, mental confusion, dry mouth, and anxiety
Deva et al. 14	Not available	No	No	Hearing loss, dizziness, and vertigo	Not available
Verma et al. 25	1/78 (1.28%): diabetes, asthma, and hypertension	Yes, without mentioning exactly how many, but stating that 2/78 individuals had to be admitted to the ICU	Yes (no details)	Bilateral reduced hearing sensitivity, speech perception problems in the presence of background noise, and mild to moderate high-frequency sensorineural hearing loss.	Total or partial loss of taste, full loss of smell, issues with tongue function and food handling during the oral swallowing phase, salivary problems (xerostomia and thick saliva), pain during swallowing, delay in completing meals, mouth odor, weight loss, change in voice quality and intensity, shortness of breath, lump sensation in the throat, tension in the neck muscles when speaking, and vocal fatigue
Saunders et al. 26	Not available	Not available	Not available	Hearing complaints	Persistent fatigue, loss of smell, memory/concentration problems, toothache, anxiety, loneliness. and despondency
Almishaal and Alrushaidan 19	39/301 (12.96%): diabetes Mellitus; 35/301 (11.63%): hypertension; 15/301 (4.98%): anemia; 14/301 (4.65%): chronic respiratory diseases; 6/301 (1.99%): cardiovascular diseases; and 2/301 (1.01%): head trauma	Yes: 103/301 (34.22%); 4/103 in the ICU. No: 198/301 (65.9%).	Hydroxychloroquine, favipiravir, dexamethasone and remdesivir	Hearing loss, ear fullness, difficulty understanding speech in silent and noisy environments, unsteadiness, dizziness, and vertigo.	Cough, shortness of breath, fatigue, fever, headache, migraine, loss of smell, loss of taste, chest pain, diarrhea, and back and joint pain
Degen et al. 18	Not available	Yes: 106/1,082 (9.80%); 26/106 (2.40%) went to the ICU	Not available	Dizziness and vertigo	Not available
Ali et al. 8	Not available	No	Neuropathic pain; alternative/supplement, antidepressant, antacid, benzodiazepine, migraine prophylaxis, beta-blocker, migraine abortifacient, sleep aid, antihistamine, anti-inflammatory, narcotic analgesic, and neuromuscular blocker	Dizziness	Brain fog, headache, numbness/tingling, pain other than chest pain, anosmia, dysgeusia, blurred vision, fatigue, depression, insomnia, shortness of breath, and chest pain
Zięba et al. 9	Type-2 diabetes (absent information on number of subjects)	Not available	Not available	Hearing disorders, dizziness and vertigo	ENT symptoms: olfactory disturbances, cacosmia, taste disturbance, headache, sore throat, runny nose, dyspnea, and cough. Systemic symptoms: weakness, fever, diarrhea, conjunctivitis, skin lesions, musculoskeletal pain, impaired memory, and movement disorders
Thrane et al. 11	Not available	Not available	Not available	Dizziness, hearing loss, sensation of a “clogged” ear, vertigo, and “false movement”	Fever, dry cough, dyspnea, fatigue, blocked nose, runny nose, sore throat, generalized body pain, headache, and chemosensory deficits
Saraf et al. 5	Not available	Not available	Hydroxychloroquine	Not available	Not available
Kartal and Kılıç 22	Not available	Yes: 40/279 (14.30%); and no: 239/279 (85.70%)	Yes: 165/279 (59.10%); and no: 114/279 (40.90%). It does not detail which medications were used	Hearing loss	Not available.,
Erinc et al. 13	3/96 (3.1%): diabetes Mellitus; 13/96 (13.5%): hypertension; and 4/96 (4.1%): thyroid gland diseases	Yes. 4/37 patients (5 to 14 days)	Not available	Hearing loss, hyperacusis, chronic otitis media and otosclerosis	Loss of smell, cough, loss of taste, shortness of breath, difficulty concentrating, increased loudness of sound and effects on life, sleep, and tinnitus-induced irritation
Espinoza-Valdez et al. 10	50/209 (23.9%): obesity; and 30/209 (14.3%): hypertension	Not available	Paracetamol, azithromycin, ivermectin, antivirals, corticosteroids, anticoagulants, steroid inhalers, hydroxychloroquine, steroid nebulizers, supplemental oxygen, tocilizumab, and chlorine dioxide	Otalgia, otitis externa, otitis media, vertigo, imbalance/instability, and hearing loss	Anosmia, hyposmia, ageusia, hypogeusia, facial pain, asthenia, headache, myalgia, fever, arthralgia, dry cough, productive cough, diarrhea, odynophagia, dyspnea, anorexia, rhinorrhea, conjunctivitis, loss of sense of smell, loss of taste, submandibular lymphadenopathy, canker sores, thyroiditis, xerostomia, facial pain, and facial paralysis
Figueiredo et al. 28	Not available	Not available	Ivermectin, azithromycin, nitazoxanide, and hydroxychloroquine	Hearing loss	Fever, fatigue, dyspnea, cough, loss of smell, parosmia, rhinorrhea, nasal congestion, myalgia, and sore throat
Khanna et al. 38	Not available	Yes (all)	Not available	Hearing loss and dizziness	Dry cough, nasal discharge, sore throat, nasal congestion, loss or alteration of sense of smell, changed sense of taste, voice changes, and throat pain
Aldè et al. 30	Not available	Not available	Not available	Vertigo, otalgia, hyperacusis, fullness earache, hearing loss, otorrhea, and dizziness	Not available
Almishaal 32	8.9%: iabetes mellitus; 8.9%: hypertension; 6.4%: chronic respiratory diseases; 1.39%: heart diseases; 0.4%: head trauma; and 5.9%: anemia	Not available	Hydroxychloroquine, favipiravir, remdesivir, and dexamethasone	Hearing loss, aural fullness, unsteadiness, dizziness, and vertigo	Cough, fatigue, sore throat, back and joint pain, headache, fever, breathing difficulties, migraine, changes in smell and taste, chest pain, diarrhea, runny nose, and sneezing
Africa et al. 34	22/187,587: heart disease; 11/187,587 type-2 diabetes mellitus; 24/187,587 hypertension; and 11/187,587: obesity	Not available	Not available	Sudden idiopathic hearing loss, sensorineural hearing loss, and vestibular disorders	Not included
Kwaśniewska et al. 33	9/346: hypertension; and 1/346: familial hypercholesterolemia	Not available	Not available	Vertigo, dizziness, sudden unilateral hearing loss, and progressive hearing loss	Smell/taste changes, dry cough, sore throat, nausea, vomiting, and dyspnea
Elmazny et al. 36	Not available	Not available	Not available	Dizziness	Fatigue, fever, headache, cough/dyspnea, anosmia/hyposmia, ageusia/hypogeusia, vomiting/diarrhea, insomnia, depression, anxiety, anosmia/hyposmia, neuropathic pain, ageusia/hypogeusia, cognitive impairment, cacosmia, and headache
Bilgin et al. 35	Not available	68 were hospitalized during the follow-up and excluded	Not available	Dizziness and otalgia	Shortness of breath, cough, chest tightness/chest pain, palpitations, weakness/fatigue, joint pain, cognitive impairment, forgetfulness, headache, insomnia, peripheral neuropathy, abdominal pain, nausea, diarrhea, menstrual irregularity, erectile dysfunction, painful urination, myalgia, osteoarthritis, anxiety, depression, loss of taste/olfaction, nasal obstruction, burning/itching eyes, and skin rash
Dorobisz et al. 37	Not available	No	No	Hearing loss	Olfactory/taste/visual changes
Borda Pedraza et al. 31	2/40 (case group): controlled hypothyroidism	Yes: 5/40 (1 in ICU); no: 35/40	Azithromycin	Vertigo, aural fullness, and hearing loss	Headache, general malaise, cough, fever, change in taste, and change in smell
Wu et al. 29	127/2,247 (5.7%): hypertension; 41/2,247 (1.8%): diabetes	Not available	No: 393/2,247 (91.82%); Yes: 35/2,247 (8.18%)	Vertigo, otalgia, ear fullness, hearing loss, otorrhea, and dizziness	Facial paralysis, cough, asthenia, fever, nasal congestion, sore throat, and runny nose

**Abbreviations:** COVID-19, coronavirus disease 2019; COVID-19 +, COVID-19-positive; ENT, ear, nose, and throat; SARS-CoV-2, severe acute respiratory syndrome coronavirus 2.

## Discussion

Based on the selected articles, the prevalence of new tinnitus varied from 0.2 35 to 96.2%, 6 while pre-existing tinnitus ranged from 8 12 to 76.2%. 5 All studies reported patient complaints about tinnitus after or during the SARS-CoV-2 infection; this occurred even though we did not include only articles that necessarily provided data on tinnitus, but articles that investigated this symptom. Moreover, it is important to mention that, despite the high prevalence of tinnitus found in the present study, there are also systematic reviews 43 that did not report tinnitus in COVID-19 patients as an ear, nose, and throat (ENT) symptom, proving the extensive heterogeneity of this manifestation.

Regarding the samples of the studies selected, there was a wide range of age groups, but most subjects were adults and/or elderly people, 2 3 4 5 6 8 9 10 11 12 13 14 16 17 18 19 20 21 22 23 24 25 26 27 28 29 31 32 33 34 35 36 37 38 the age groups with the most cases of tinnitus, especially older individuals. Therefore, this symptom is related to aging, since 90% of tinnitus cases have hearing loss as their main cause, a common disease in old age and sometimes with tinnitus as a symptom. 44

Approximately half of the studies 2 6 7 10 13 15 16 17 19 23 25 27 29 31 32 33 34 reported subjects with comorbidities concomitant with SARS-CoV-2 infection, likely related to the aging process *.* This may reveal that tinnitus could be influenced by diseases such as SAH, a predominant finding in the current study 2 6 7 10 13 15 16 17 19 23 25 27 29 32 33 34 and a possible cofactor or aggravator of preexisting factors in the generation of this symptom 45 In addition to hypertension, other comorbidities may overlap as possible causes of tinnitus. Therefore, assessing if these are isolated causes of the symptoms became difficult due to the global scenario during the pandemic, making it impossible to previously investigate tinnitus in studies, since the disease began acutely and widely throughout the world.

As for other hearing complaints, there was a prevalence of hearing alteration/loss, 2 3 4 6 7 9 10 11 12 13 14 16 17 19 20 22 23 24 25 26 27 28 29 30 31 32 33 34 37 38 which is in line with the literature, since evidence reveals possible damage to the sensory and mechanical structures of the auditory system in patients affected by COVID-19. 46 The SARS-CoV-2 infection may affect the functions of the inner hair cells of the organ of Corti, structures responsible for activating afferent receptors in response to pressure waves that reach the basilar membrane through sound transduction, leading to hearing loss, usually sensorineural. 27 As for extra- auditory symptoms, there was a predominance of olfactory 2 3 6 7 9 10 12 13 15 16 17 19 23 25 26 27 28 31 32 33 35 36 37 38 and taste disorders, 2 3 6 7 9 10 12 13 15 16 17 19 23 25 27 31 32 33 35 36 37 38 followed by cough 2 3 9 10 11 12 15 16 17 19 27 28 29 31 32 33 35 36 38 and fever. 2 3 9 10 11 12 15 16 19 27 28 29 31 32 36

The presence of new tinnitus was reported in 33 studies, 2 3 5 6 7 8 9 10 11 14 15 16 17 18 19 20 21 22 24 25 26 27 28 29 30 31 32 33 34 35 36 37 38 while 11 articles 4 5 12 13 16 19 22 23 26 27 28 revealed changes (worsening and/or improvement) in preexisting tinnitus, showing that SARS-CoV-2 infection has a likely relationship with the pathophysiology of this symptom, both for its onset and severity changes. As aforementioned, the prevalence of tinnitus in the studies was variable due to the high variability of the samples, ranging from 26 6 to 375,174 34 subjects. Moreover, four articles included children, 7 15 30 38 which can lead to variations in the way the onset and effects of tinnitus are found and interpreted.

It is not feasible to establish a direct correlation between tinnitus and COVID-19, given the vulnerability of chronic tinnitus to psychological burdens and/or emotional stress. Therefore, the fragility triggered by the COVID-19 pandemic can be seen as the stressor. 13 On the other hand, there is the possibility of having the SARS-CoV-2 virus spreading in the body, interacting with the nervous system 23 and generating inflammation of the cranial nerve eight, besides symptoms such as tinnitus, deafness, and vertigo. 47 The virus can also reach the middle ear via the auditory tube, leading to inflammation, obstruction, and auditory and vestibular symptoms. 46

Likewise, the use of medications to deal with COVID-19 symptoms has been the subject of research into their effects on the auditory and vestibular systems, especially hydroxychloroquine, 48 which was used by individuals in five studies included in the present review. 5 10 19 28 32 Evidence found in the literature reveals that the use of ototoxic drugs can increase the prevalence of tinnitus, 48 49 making it impossible to confirm a direct association between tinnitus and COVID-19.

Regarding the traits of tinnitus, data was scarce in the articles, since most studies did not investigate details of the symptoms. Incomplete information stands out, especially when describing relevant tinnitus factors, such as duration, onset, laterality, reversibility, and aggravation, among others.

More, we should emphasize that only the articles with information on preexisting tinnitus 4 5 12 13 16 19 22 23 26 27 28 included assessments such as the simplified version of the Tinnitus Handicap Inventory (THI-S) to estimate the symptom before SARS-CoV-2 infection. In new cases of tinnitus, the tests were only conducted postinfection and the onset of the complaint, with no previous results to draw a comparison, as recommended by the American Academy of Audiology. 50

Pre-COVID-19 evaluations are extremely important for an adequate visualization of the factors that cause the onset of tinnitus. However, the sudden onset of calamity caused by the pandemic made it difficult and/or impossible to perform objective and subjective exams due to issues of social isolation. Therefore, we stress the importance of animal model studies to establish cause-and-effect relationships by excluding causal factors and avoiding possible biases. Yet, we highlight the need to question patients about the presence of tinnitus in routine consultations and of carrying out investigative tests when tinnitus is reported. New studies that investigate tinnitus longitudinally through reliable tests are essential and should be encouraged.

Still on the importance of carrying out objective tests on complaints of tinnitus, specific exams such as threshold tonal audiometry and tympanometry are essential to determine the nature of tinnitus, which can also occur due to changes in the middle ear. However, among the 37 studies included, only 9 5 6 13 20 24 28 30 31 37 mention the performance of these tests at some point during data analysis, while, the other 28 2 3 4 7 8 9 10 11 12 14 15 16 17 18 19 21 22 23 25 26 27 32 33 34 35 36 38 39 articles determine the occurrence of the symptom through self-reporting, commonly through online questionnaires. This probably occurred because several studies were carried out during the COVID-19 pandemic, making it difficult to perform these tests due to contact prevention measures; however, it is essential that future studies include these tests to avoid possible bias.

Hence, based on the GRADE system, 41 there is a low level of evidence, considering that all articles are observational studies (classified in category C). According to the classification of the Oxford Centre for Evidence-Based Medicine, 40 only two studies 16 24 were classified as presenting level two of evidence. Five articles 8 12 13 20 37 was allocated to level three, and the others were classified as level four, 2 3 4 5 6 7 9 10 11 14 15 17 18 19 21 22 23 25 26 27 28 29 30 31 32 33 34 35 36 38 proving the reduced evidence of most studies.

Considering this context, a limitation is found on the fact that articles classified as presenting level two of evidence contain inconsistencies, since the scientific design mentioned in their methodology proves to be incompatible with the design proposed in their development. Both call themselves cohort studies, but they do not present measurements over a period in a group of participants identified at the beginning of the study, 51 as this type of design demands.

Yet, the present research has other important limitations that can generate bias. The absence, in most studies, of pre-COVID-19 evaluations, which probably occurred due to the unpredictable nature of the disease and the need for social distancing brought by the pandemic, made the longitudinal and detailed analysis of the symptom inaccurate. Moreover, the small number of studies that included objective tests such as threshold tonal audiometry or tympanometry made it hard to determine the nature of tinnitus and, consequently, exclude possible middle ear factors that may be co-occurring with SARS-CoV-2 infection. Despite this bias, analysis of this symptom needs to be carried out to guide new studies conducted outside the context of the pandemic and with adequate methodological rigor.

There is a clear need for new studies that produce results with high scientific evidence, capable of isolating tinnitus and SARS-CoV-2 infection from other comorbidities and toxic medications. Since our findings are mostly based on patient self-reports, more detailed and objective research is essential to assess if these symptoms are due to COVID-19, the effects of medications used, middle ear issues, previous diseases, or stress factors during the illness process. The fact that we were not able to determine whether there is a direct correlation between tinnitus and SARS-CoV-2 is the one of the biggest limitations of the current study. This is needed to understand the real impact of the disease on the symptom's physiopathology, and it is also necessary to conduct systematic reviews with meta-analyses based on future studies designed with greater methodological rigor, aiming to demonstrate such correlations statistically.

## Conclusion

The prevalence of new tinnitus ranged from 0.2 to 96.2%, while pre-existing tinnitus ranged from 8 to 76.2%. Nonetheless, we could not satisfactorily analyze the characteristics of tinnitus. Therefore, a direct correlation between tinnitus and COVID-19 cannot be determined, since this symptom may be influenced by other factors, which may present themselves as possible stressors, and not necessarily the contamination by the virus.
